# Surface Electrochemistry of Au(111) in Acetonitrile Based Electrolytes: Formation of a Solvent Related Adsorbed Layer

**DOI:** 10.1021/acs.jpclett.6c01308

**Published:** 2026-07-08

**Authors:** Greta P. Grossman, Milena Martins, Alenka Krizan, Andrijana Marojevic, Jan Bitenc, Dusan Strmcnik, Marc T. M. Koper

**Affiliations:** † Leiden Institute of Chemistry, 4496 Leiden University, Einsteinweg 55, 2333 CC Leiden, The Netherlands; ‡ 68913 National Institute of Chemistry, Hajdrihova 19, 1000 Ljubljana, Slovenia; § Faculty of Chemistry and Chemical Technology, University of Ljubljana, Večna pot 113, 1000 Ljubljana, Slovenia

## Abstract

Fundamental understanding of the electrochemistry at metal/nonaqueous solvent interfaces is of importance both for improving organic solvent based electrochemical devices and also for generalizing theories of interfacial electrochemistry that were developed based on aqueous solvents. Herein we report electrochemical characterization of the Au(111)/acetonitrile (ACN) interface in verifiably clean conditions, in a potential region normally considered nonreactive or ideally polarizable. We demonstrate the formation of a solvent related adsorbed layer on the Au(111) surface upon contact with the electrolyte, which further undergoes oxidative and reductive transformations in subsequent cyclic voltammetry. The presence of this layer is further confirmed by *ex situ* electrochemical characterization and X-ray photoelectron spectroscopy. We also show how the composition of the adsorbed layer depends on the electrolyte composition and the applied electrochemical program. Taken together, these results show that the adsorbed layer incorporates both (decomposed) acetonitrile and electrolyte ions and that the Au(111)/ACN interface cannot be considered ideally polarizable. Our findings represent a significant step toward a more precise understanding of the interfacial electrochemistry of the Au(111)/ACN interface and demonstrate the importance of interphase layers in the general understanding of nonaqueous surface electrochemistry.

## Graphical abstract

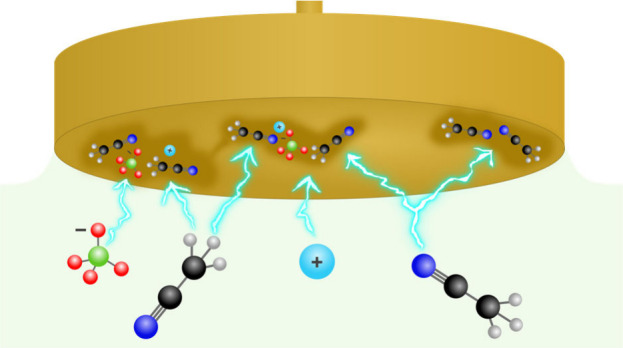

Applications of electrochemical systems based on organic solvents such as batteries,^1,2^ supercapacitors^2^ and organic electrosynthesis,^3^ are technologically advanced. However, the interfacial electrochemistry in these systems is often still poorly or incompletely understood. In particular, there are limited studies focusing on surface electrochemistry in the absence of charge-transfer processes that elucidate the structure of the corresponding electrochemical double layer (EDL). There are two broad approaches in interpreting interfacial electrochemical behavior in nonaqueous electrolytes. Most of the older work uses the classical Gouy–Chapman–Stern (GCS) model to interpret EDL capacitance curves, in a very similar way to how it has been used for aqueous interfaces.^4–11^ On the other hand, a few recent studies have brought forward significantly different ideas, suggesting the formation of a solvent related interphase layer at various organic solvent/metal interfaces.^12,13^ Such a process would dominate the electrochemical response such as the differential capacitance.

EDL properties of several solid metal/nonaqueous solvent interfaces have been studied, in particular in the 1980s and 90s. Capacitance curves have been measured using electrochemical impedance spectroscopy (EIS) or alternating current cyclic voltammetry (ACV) at frequencies between 20 and 1000 Hz. These curves generally show the capacitance minimum and the typical concentration dependence predicted by GCS theory.^4–11^ Several of these capacitance curves have also been reproduced by continuum modeling,^4,14,15^ and these results have led to explanations for some solvent related differences in capacitance. Despite this alignment, several electrochemical features of these interfaces have proven to differ significantly from expectations based on GCS theory and aqueous interfaces. Shapes of cyclic voltammograms (CVs), when reported,^5,7^ do not match capacitance curves, suggesting that processes other than EDL capacitance could be present outside of the explored frequency range. Capacitance curves have also been shown to evolve with time and differ based on the potential window explored for several different nonaqueous interfaces,^6–9^ which hints at the existence of an additional time-dependent surface process. Parsons–Zobel plots often have slopes lower or higher than 1, or deviate from linearity.^5,7,8^ Solvent adsorption has been suggested as one possible explanation for these discrepancies.^6,7,10^ Indeed, chemical and physical interactions between the metal and solvent likely differ significantly for each solvent/metal pair and would be expected to play a major role in determining the properties of the interface and the EDL.

It is well-known in the battery community that a solid electrolyte interphase (SEI) often forms between a metal or carbon electrode and an organic electrolyte. SEIs form due to electrochemical interactions between the solvent, electrolyte ions, and the electrode, in particular through potential-dependent solvent decomposition at relatively large reductive potentials.^16^ Chen et al.^12^ have recently observed the formation of a similar “soft layer” at the interface between gold, copper and carbon electrodes and various organic solvents at mild potentials near the presumed potential of zero charge (PZC). This soft layer, however, has properties that make it distinct from SEIs and the authors suggest that their findings call for a new understanding of electrochemistry at metal-nonaqueous solution interfaces. There are also other reports of interphase layers at nonaqueous electrochemical interfaces. Chen et al.^13^ observed overlayers containing tetrabutylammonium (TBA^+^) and decomposition products of acetonitrile (ACN) at Pt/ACN interfaces detected by surface-enhanced infrared absorption spectroscopy (SEIRAS). Gunasekaran et al.^17^ reported the formation of colored films and IR bands corresponding to ACN decomposition products at the Au/ACN interface below −1 V vs Ag/Ag^+^. These authors also suggested that ACN forms a chemical bond with the Au surface based on SEIRAS,^17^ in agreement with earlier results by Reinsberg and Baltruschat.^18^ However, it is not clear from these studies if this chemical bonding results in a permanently modified electrode surface with ACN related adsorbates remaining on the surface even after electrochemical treatment. Although it is not known how general the occurrence of chemically modified interphase layers is for metal/nonaqueous solvent interfaces, there is compelling evidence for their presence in at least some cases with various different experimental conditions.^12,13,17^ It is also not currently clear to what extent the interphase layers mentioned above are similar to SEIs, which are known to form in more complex battery systems at more extreme potentials. A chemically modified interphase layer will undoubtedly influence EDL properties and the corresponding interfacial electrochemistry. In order to gain a better understanding of these effects, here we focus on investigating the formation mechanism, electrochemical behavior and composition of such an interphase layer at a well-defined single-crystalline electrode in a highly common organic solvent, namely the Au(111)/ACN interface.

For many aqueous electrochemical interfaces, molecular scale understanding of interfacial electrochemistry and EDL properties has been achieved to a good level of detail.^19–24^ The use of “blank” CVs of single-crystal electrodes has been an important enabler of this progress.^19^ These CVs can be used to demonstrate cleanliness as well as to identify and track specific surface processes which correspond to peaks in the CV. A similarly detailed understanding of surface electrochemistry, in particular of the correspondence between surface processes and CV peaks, has not yet been achieved for organic electrolytes. Toward this goal, we show here CV characterization of Au(111) in ACN-based electrolytes in demonstrably clean conditions and present an initial interpretation of the CV features in terms of specific surface processes. We also present electrochemical and spectroscopic evidence for the formation of a solvent related adsorbed layer.

To ensure that surface processes were captured accurately and to minimize interference from impurities, a rigorous standard of cleanliness and exclusion of water was adopted for all experiments (see Supplementary Notes 1 and 2 for details). Briefly, all experiments were performed in an Ar filled glovebox with <0.1 ppm of O_2_ and H_2_O. ACN was dried with molecular sieves and fractionally distilled before use. The purity of the solvent was verified by gas chromatography–mass spectrometry (GC–MS) (Figure S1). Water content of the electrolyte was determined by Karl Fischer titration to be <1 ppm both before and after electrochemical experiments. Glassware was cleaned in an acidic KMnO_4_ solution followed by a dilute solution of H_2_SO_4_ and H_2_O_2_ and repeated rinsing and boiling with ultrapure water. Traces of water were removed by drying in a vacuum oven. Salts were dried in a mini vacuum oven inside the glovebox. An Ag wire was used as a pseudo reference electrode, to avoid contaminations from commonly used leakless Ag/AgCl reference electrodes. As shown in Figure S3, when the Ag wire is replaced by a leakless Ag/AgCl reference electrode an additional large reduction peak appears (presumably related to chloride desorption) and the original CV features are suppressed. These changes get worse over time after the immersion of the Ag/AgCl electrode. Due to the extremely high sensitivity of the surface processes reported here, these procedures are necessary to observe the reported electrochemical features. The effect of relaxing the standards of cleanliness is exemplified by Figure S4, which shows that the CV is clearly impacted when the experiment is performed outside of the glovebox, even when all other experimental parameters remain unchanged.

Figure 1a) shows the first 15 cycles of the CV of Au(111) in ACN with 1 mM TBAClO_4_. The main feature in this CV is a sharp oxidation peak around 0.87 V vs Ag/Ag^+^ with a corresponding reduction peak at ca. 0.75 V vs Ag/Ag^+^. We assign the oxidative peak to a transformation in an adsorbed layer (and perhaps partially oxidative adsorption of the layer itself) and the reductive peak to a (partial) reduction or desorption. A flat oxidative feature is present between [−0.2;0.3] V vs Ag/Ag^+^ (Figure 1a), which is sometimes partially or fully replaced by a sharper peak at ca. 0.6 V vs Ag/Ag^+^ (such as in Figure 1b). This feature possibly corresponds to an initial oxidation/adsorption step that precedes the main oxidation event at 0.87 V. Finally, a small reductive feature is present around −0.25 V vs Ag/Ag^+^, which may be related to an additional desorption step or a reduction of the species in the adsorbed layer formed on the surface. All peaks apart from the large anodic peak in the first cycle have irregular shapes, indicating that they consist of multiple processes. It is typical of gold single crystals to have adsorption processes consisting of several steps corresponding to the formation of different surface layers and showing up as multiple overlapping peaks in the CV.^22^ Although we currently cannot provide a detailed assignment of all the overlapping processes, a few notable features are highlighted here.

**Figure 1:**
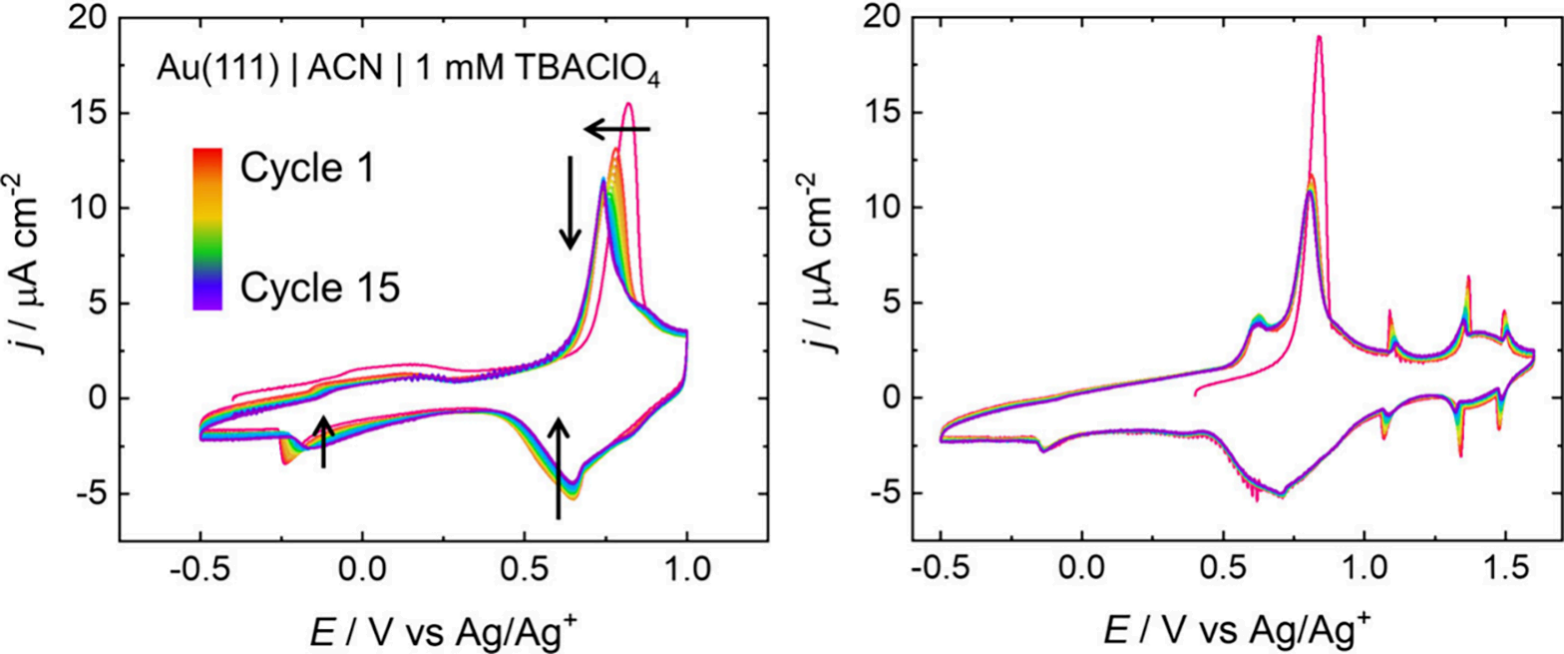
CV characterization of Au(111) in ACN with 1 mM TBAClO_4_ at 50 mV s^–1^ with a) showing the first 15 cycles between [−0.5;1] V vs Ag/Ag^+^ b) the first 10 cycles of the CV between [−0.5;1.6] V vs Ag/Ag^+^.

Both the oxidation and reduction currents decrease gradually with increasing cycle number, although the extent of the decrease depends strongly on conditions, as is e.g. evident from the comparison between Figure 1a and 1b. This continuous change when cycling indicates that the interface is not at steady state and is slowly evolving during the entire experiment. As the cycle number increases the change from cycle to cycle gradually gets smaller, suggesting that a steady state is slowly approached. We interpret this gradual change in the CV currents as a slow buildup/transformation of an adsorbed layer, such that the surface is slightly different at the beginning of each cycle. The decreasing currents can be interpreted as a lower extent of continuous surface modification or adsorption on the increasingly modified surface. Steady state is reached once the currents in each subsequent cycle are the same, presumably indicating that the adsorbed layer is fully saturated and no more adsorbates can be accommodated.

To approximate the charge associated with the adsorbed layer, we assume that transformation of adsorbed species happens in the oxidative scan during the large peak at 0.87 V vs Ag/Ag^+^, followed by reduction or partial desorption in the reverse peak during the negative-going scan. Thus, the evolution of the species transformed in each cycle can be approximately associated with the charge density under the main oxidative and reductive peaks (Supplementary Note 3.1). The amount of charge in both directions shows an overall decreasing trend as shown in Figure S5, indicating that the electrochemical reactions in both directions proceed to a lesser extent as the cycle number increases. The total charge per cycle is between 20 and 30 μC cm^–2^, which corresponds to ∼1/8 of a monolayer on a Au(111) surface.^25^ Although it is unclear if we can interpret this process in classical terms of filling up adsorption sites, the value of the total charge indicates that only around 1 out of 8 Au atoms forms a bond with an adsorbate and/or that less than one electron is transferred during the adsorption process. This is a reasonable value for ACN adsorbates which may be arranged in a flat-lying or tilted configuration on the Au(111) surface, in which case they would take up more than a single Au atomic site. Partial charge transfer between Au(111) and ACN is also plausible, for instance via the sharing of the N lone pair from the ACN molecule.

Figure S6 shows the capacitance values calculated from the first cycle of CVs taken with various scan rates between 5 and 300 mV s^–1^. The peak to peak separation for CVs with different scan rates increases with increasing scan rate (Figure S7), indicating a kinetically limited quasi-reversible reaction.^25^ The capacitance values of the main oxidation peak show reasonably good overlap for the different scan rates, which is further confirmed by Figure S8, demonstrating a direct proportionality between the scan rate and the oxidative peak current. This direct proportionality indicates an electrochemical process limited by available surface sites, not diffusion, demonstrating further agreement with a surface-confined process.

Figure 1b) shows the CV of Au(111) in the same solution with an extended potential range. Three sharp reversible redox peaks are visible in the potential region between [1;1.5] V vs Ag/Ag^+^. The sharpness of the peaks decays slightly with increasing CV cycles. All three of these redox peaks have peak to peak separation significantly below 57 mV (Table S1), which demonstrates that they must originate from surface processes.^25^ These peaks appear very similar in shape, position and reversibility to the well-known butterfly peaks observed on Au(111) in H_2_SO_4_, which are known to arise from a phase transition in the adsorbed adlayer (Figure S9 and Table S1). Ordered layers of aromatic compounds adsorbed from aqueous solutions^26–28^ as well as surface layers formed in solvent free ionic liquids^29^ have been observed to give rise to such sharp CV peaks on Au(111), and were shown to be related to phase transitions within the layer. This correspondence suggests that a similar phase transition-type process in the adsorbed layer may happen in our case.

The CV in Figure 1 does not show any EDL related features as predicted by GCS theory, such as the characteristic camel-shaped CV peaks and capacitance minimum which are known to arise on Au single crystal surfaces in aqueous electrolytes.^20,30^ If these processes do occur, the corresponding currents are likely much smaller than the adsorption currents we have identified and thus the EDL peaks might be present but not visible. However, CVs shown here characterize an already modified surface that may not behave according to GCS theory, which is strictly applicable only to unmodified ideally polarizable surfaces. As demonstrated by Figure S10, modification of the electrode surface starts happening at the beginning of the applied potential program, regardless of the applied potential, so that the first CV cycle already shows a partially modified surface. It is likely that GCS theory alone will not be able to explain double layer behavior at these modified surfaces.

Figure S10 also demonstrates that surface modification, and therefore adsorption takes place in the entire potential range, not only during the main oxidation peak at 0.87 V vs Ag/Ag^+^. This means that a Au(111) surface subjected to any electrochemical treatment in the present electrolyte is expected to be modified in some way. The oxidation peak at 0.87 V vs Ag/Ag^+^ is the dominant charge transfer event in the examined potential range. However, the exact relationship between the observed CV peaks and the formation/transformation of the adsorbed layer is not straightforward and we cannot currently provide a comprehensive description.

There are very few comparable CVs reported in the literature characterizing Au electrodes in ACN solvent in a comparable potential range without interference of other reactions. However, our CV does show some similarity to the CV of polycrystalline Au in ACN with 100 mM TBAClO_4_ reported by Ledezma-Yanez et al.^31^ A large oxidative peak that decreases with subsequent cycles is also reported in this study at a similar potential to our main adsorption peak.

We note that the Au(111) surface is well established in literature to exhibit the (22 × $\sqrt{3}$ ) herringbone reconstruction at negative potentials (either thermal or potential-induced), while the reconstruction is lifted at positive potentials.^20,32–34^ The process of lifting and re-forming the reconstruction can be slow and its exact potential range depends on the extent of adsorption of the electrolyte species. It has been shown that the (lifting of) the reconstruction occurs in virtually every electrochemical condition, including with large adsorbed organic molecules^35^ and in solvent-free ionic liquids,^36^ therefore it is likely present in our case. The lifting of the thermal reconstruction on Au(111) is manifest by a sharp oxidative peak in the CV when strongly adsorbing species, such as SO_4_^2–^, are present.^32^ It may be the case that the lifting of the reconstruction contributes to the sharp peak we have observed at 0.87 V vs Ag/Ag^+^ in the first cycle. However, such a sharp peak is generally only observed during the lifting of the thermal reconstruction during the first cycle. One additional possibility that could (partially) explain the cycle number dependent changes (as well as the starting potential dependent differences in Figure S10) would be a slow, partial lifting of the reconstruction, which takes many cycles to complete. A more in-depth study of the surface reconstruction in our system using *in situ* techniques would be needed to reveal the details of this process.

To investigate the sensitivity of the blank CV to electrolyte composition, we performed CV experiments with electrolytes with various ionic composition, salt concentration and water content. Figure 2a) shows a comparison between 1 mM TBAClO_4_, LiClO_4_ and TBAPF_6_ electrolytes. All three CVs have the characteristic oxidation and reduction peaks and show the characteristic current decay during cycling (Figure S11). The main redox features appear to be largely ion insensitive and have very similar shape and position for all three electrolytes. These similarities demonstrate that the main species interacting with the surface and responsible for the CV features cannot be the cation or the anion and thus must be the solvent.

**Figure 2:**
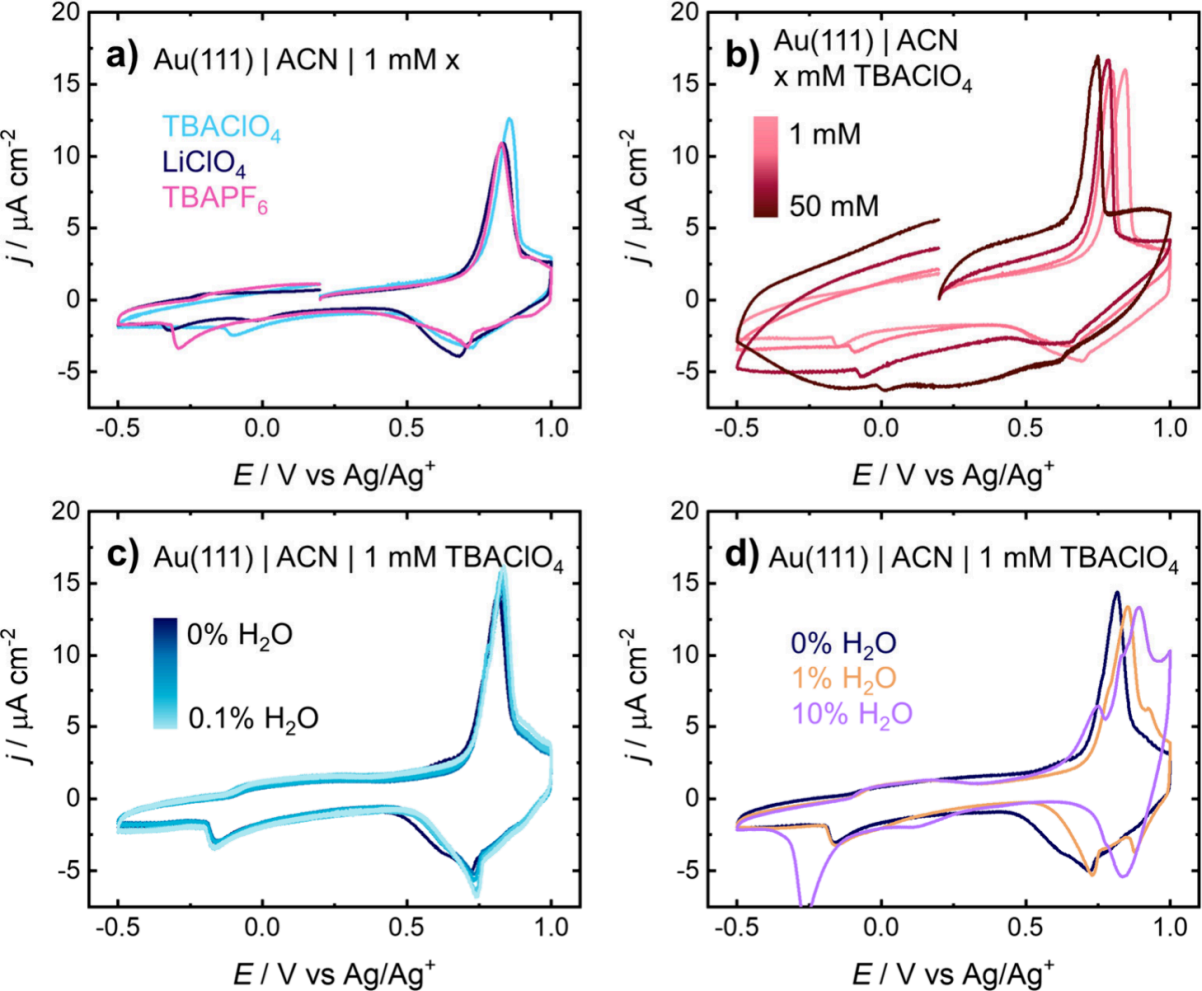
CV of Au(111) in ACN at 50 mV s^–1^ showing a) the effect of changing the anion or cation on the first cycle, b) the effect of increasing the concentration of TBAClO_4_ on the first cycle, c) and d) the effect of increasing water content on the 10^th^ cycle. Water contents are given as volume %.

Figure 2b) shows CVs with different concentrations of TBAClO_4_. Although the salt concentration clearly affects the CV shape, the main adsorption and desorption peaks are clearly identifiable at every salt concentration. This is consistent with the idea that the solvent is the main adsorbing species responsible for the CV features. Increasing the salt concentration has three main effects on the shape of the CV. First, the larger oxidative and reductive peaks both move to more negative potentials, while the smaller reductive peak moves to more positive potentials. Since a pseudoreference electrode was used, it is possible that a shift in the reference electrode potential with increasing salt concentration is responsible for one of these shifts; however, it cannot account for the shift of two peaks in different directions. This potential shift suggests that the electrolyte ions have some type of modulating effect on the adsorption process. Second, the peaks evolve and decay more rapidly for higher ionic concentrations (Figure S12), implying that the adsorbed layer saturates faster. Third, the CV also has more capacitive background current for higher ionic concentrations, which may suggest enhanced ion incorporation into the adsorbed layer and formation of a thicker or denser layer with more capacitive charge. At the same time, Figure 2b) shows no evidence of the characteristic GCS-like changes with concentration,^25,37^ suggesting that EDL capacitance cannot be explained by traditional models.

Figure 2c) and d) show CV curves with increasing amounts of water added. Surprisingly, additional water has very little effect on the CV up to 0.1 volume % (equivalent to 44 mM or 1000 ppm). The only clearly identifiable change is a slight sharpening of the main reduction peak with increasing amounts of water. With 1% and 10% of water the CV does appear significantly distorted. These results suggest that there are two distinct concentration regimes for water. At low water concentration, ≤0.1%, water does not seem to be present in the interfacial region and does not affect the shape of the voltammogram. In the concentration range ≥1% water enters the interfacial region and has a measurable effect on electrochemical processes.

We suggest a possible rationalization of this two-domain behavior based on the well-established microheterogeneity of ACN/water mixtures.^38–42^ In ACN solutions with intermediate to high water contents, water molecules preferentially form H-bonded clusters rather than bond with ACN molecules. This behavior persists when ions are present and is also expected to extend to the interface.^39^ Several studies have demonstrated that solvent microheterogeneity can have a significant impact on electrochemistry when water contents are around 2.5% or above.^39–43^ Presumably this is the type of water behavior responsible for the high water contents in Figure 2d). As water contents get lower there are too few water molecules to form H-bonded clusters and water molecules are either isolated or form oligomeric aggregates.^44,45^ The shift in the electrochemical behavior of water we have observed here between 0.1 and 1% might reflect this tipping point in the structure of ACN/water mixtures.

To confirm that the Au(111) surface has been modified by an adsorbed layer after CV in ACN, we performed two *ex situ* tests on Au(111) electrodes that have been electrochemically treated in ACN based electrolytes. After electrochemical treatment, the electrodes were rinsed with 300 μL ACN to wash away the droplet formed after removal of the electrode from the electrolyte solution. ACN from the rinsing procedure was left to evaporate.

We first tested the electrochemical properties of the modified Au(111) surface by running a CV in an aqueous HClO_4_ solution. For this experiment the Au(111) electrode was cycled 15 times in an ACN electrolyte with the same parameters as the CV shown in Figure 1a). After washing with ACN, the electrode was transferred into a new cell outside the glovebox, containing 1 mM HClO_4_ aqueous electrolyte. The first 15 CV cycles of the modified Au(111) in 1 mM HClO_4_ are shown in Figure 3, alongside the CV of a freshly annealed Au(111) electrode in the same solution. The original GCS-like capacitance features of Au(111) completely disappear after treatment in ACN, showing that the electrochemistry of the modified surface does not follow GCS theory, even on a qualitative level. The modified Au(111) electrode shows a strong oxidative wave starting around 0.75 V vs RHE and two reductive peaks around 0.8 and 0.5 V vs RHE, which are seemingly related to the large oxidation feature in the positive-going scan. From the corresponding charges, which are similar to the charges of the redox peaks in ACN, these features can likely be assigned to the oxidation and reduction of species contained in the adsorbed layer formed from modification in ACN. Subsequent CV cycles show gradual changes, with the large oxidation wave decreasing slightly in each cycle. This suggests that the adsorbed layer is relatively stable in the aqueous electrolyte with only slow degradation over time.

**Figure 3:**
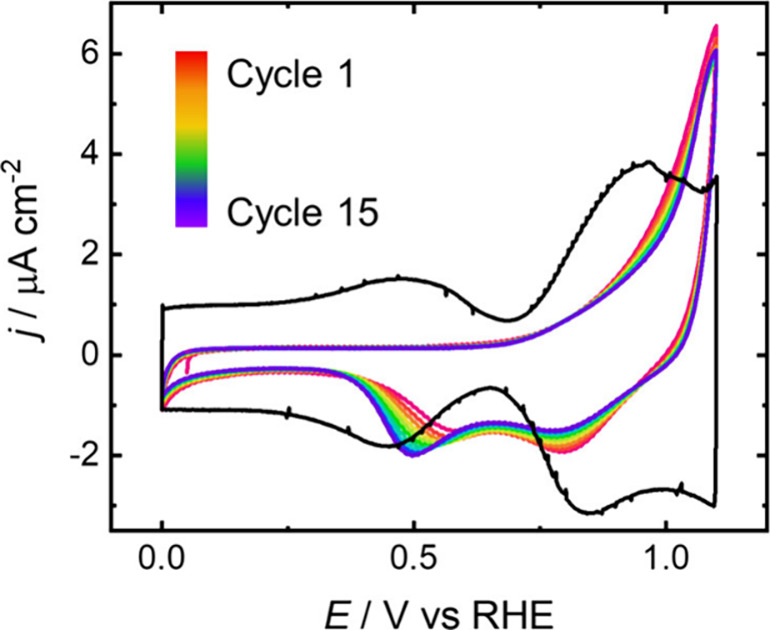
CV of a freshly annealed Au(111) (black) in 1 mM HClO_4_ and in the same solution, first 15 cycles of the CV of a Au(111) electrode previously treated with 15 CV cycles in ACN with 1 mM TBAClO_4_ (rainbow colors), both at 50 mV s^–1^.

To probe the composition of the adsorbed layer, we performed X-ray photoelectron spectroscopy (XPS) on Au(111) electrodes electrochemically treated in ACN electrolyte. LiClO_4_ was used instead of TBAClO_4_ for all of these samples to ensure that the N 1s spectra had contributions only from ACN. Au(111) electrodes treated in four different ways in ACN electrolyte with 1 mM LiClO_4_ are compared. Sample (i) was subjected to 50 CV cycles (Figure S13) and washed with ACN. Sample (ii) was held at 0.2 V vs Ag/Ag^+^ (starting potential for the CV of sample (i)) for 50 min (the same duration of time as the CV treatment of sample (i)) and then washed with ACN. Sample (iii) was immersed in the same electrolyte for the same duration of time with an open circuit, with no current flow in the external circuit, and then washed with ACN. (Open circuit potential [OCP] was measured to be around 0 V vs Ag/Ag^+^ and was seen to slightly evolve over time.) Sample (iv) was placed in the XPS chamber directly after annealing without any treatment in ACN.

XPS survey spectra (Figure S14) show the expected peaks from C, N, O, Cl, Au and Li and no additional peaks. However, due to interference from adventitious carbon and overlaps in XPS binding energies (BEs) only the N 1s and Cl 2p spectra can be reliably used for analysis (further discussion in Supplementary Note 5). Figure 4 shows high resolution XPS spectra of these BE regions for samples (i)–(iii). Sample (iv) showed no visible peaks in these two regions, confirming that the freshly annealed surface was free of N and Cl containing species (Figure S18). For the full set of spectra and analysis details see Supplementary Note 5.

**Figure 4:**
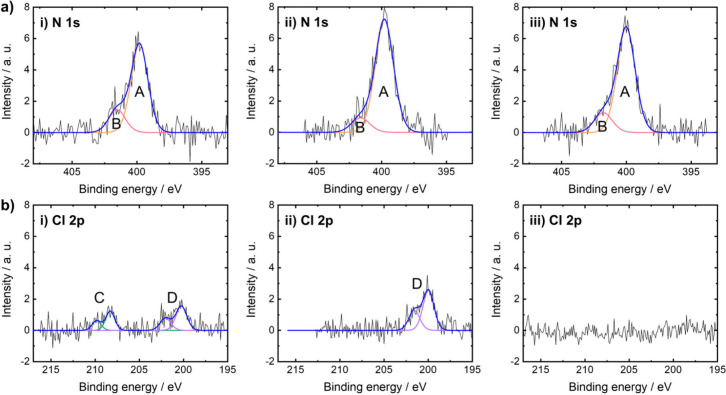
High resolution XPS spectra of the a) N 1s and b) Cl 2p spectra of a Au(111) single crystal surface subjected to various forms of electrochemical treatment in ACN with 1 mM LiClO_4_, specifically (i) 50 CV cycles between [−0.5;1] V vs Ag/Ag^+^, (ii) 50 min holding the potential at 0.2 V vs Ag/Ag^+^ and (iii) 50 min of immersion at OCP.

N 1s species detected on samples (i)–(iii) demonstrate the presence of an adsorbed layer that contains (fragments of) ACN and remains on the Au(111) surface after washing as a result of various types of electrochemical treatment. The N 1s peak is similarly shaped on all three samples and requires at least two components to fit, implying that ACN is present in two distinct chemical states or binding modes. Based on the large full width half maximum (FWHM) values (Table S2), it is possible^46^ that the peaks consist of even more components. The BE for the larger N 1s component (A) is similar for all three samples, between 399.6 and 400 eV, which corresponds well with expectations for ACN adsorbed on Pt^47^ and cyanide species in general.^48^ Surface bound ACN was previously reported to have a negatively shifted BE;^47^ however, the smaller N 1s component (B) in our sample is found at a considerably higher BE, between 401.3 and 402 eV. This uncharacteristically high N 1s BE suggests that ACN is not simply coordinated to the surface via the N lone pair but instead involved in a type of bonding that leaves the N atom more electron poor, possibly a positively charged N atom. Based on Figure 4a) and Table S2 we can conclude that samples (i)–(iii) contain similar amounts of adsorbed ACN with broadly similar electronic structure. It should be noted, however, that similarly shaped XPS spectra do not necessarily imply identical chemical compositions, since chemically distinct species may have very similar BEs.^46^

The Cl 2p region on the other hand looks distinctly different depending on the form of electrochemical treatment; ClO_4_^–^, and therefore likely also Li^+^, are only present in the adsorbed layer on samples (i) and (ii). The apparent absence of ionic species from the interphase layer on sample (iii) may suggest that the OCP potential we measured is close to the PZC of this interface. Two distinct (doublet) peaks are present on the electrochemically cycled sample (i) at 208.2 and 200.2 eV, while one species at 200.0 eV is visible on sample (ii). The peak at 208.2 eV aligns well with the expected BE for ClO_4_^–^,^48,49^ while the peak around 200 eV can be tentatively assigned to a C–Cl species, although its BE is slightly higher than reported values.^48,50^ This lower BE Cl 2p peak suggests that ClO_4_^–^ participates in a chemical or electrochemical reaction resulting in the formation of C–Cl or other [x]-Cl species in samples (i) and (ii). This would be quite an unexpected observation since ClO_4_^–^ is generally considered to be a stable and unreactive electrolyte ion. The significant differences in the ClO_4_^–^ species on the different samples suggest that at least the ionic component of the adsorbed layer is dependent on the applied potential program.

In conclusion, we have reported the blank CV of a Au(111) single-crystal electrode in acetonitrile electrolyte in verifiably clean conditions. Our findings provide experimental evidence that during electrochemical treatment, acetonitrile interacts physically and/or chemically with the electrode surface and forms an adsorbed organic layer which also includes ionic species. This adsorbed layer chemically modifies the electrode and fundamentally changes the nature of the interface. The exact composition of this adsorbed layer is influenced by the applied potential program, but modification of the electrode occurs after various different forms of electrochemical treatment. Even at mild potentials and with low concentrations of electrolyte, the CV shows no GCS-like capacitance behavior, instead the electrochemical profile of the “blank voltammetry” is dominated by oxidation and reduction processes associated with an adsorbed layer.

A number of open questions remain concerning the nature of the solvent related adsorbed layer and its interfacial electrochemical characteristics:

- The exact nature of the chemical and/or physical driving force for the interactions between the Au(111) surface, ACN and ionic species remains unresolved. It is also unclear to what extent the solvent molecule is chemically altered during the adsorption.
- Our findings suggest that water content has little effect on electrochemistry below about 0.1%. Although we have suggested some possible reasons for this, it is an unexpected result and further studies in this low water range will be needed to provide a more certain explanation.
- It is likely that chemical modification of the electrode affects Faradaic processes at this interface. How exactly different charge-transfer processes are affected and how the adsorbed layer itself might change during reactions remains a question for further research.
- Interestingly, our results show no alignment with expectations based on GCS theory, even on a qualitative level. At the same time, the adsorbed layer contains ionic species that are confined at the surface, which will participate in screening the electric charge in the EDL. It will be the subject of further studies to determine how these surface confined ions affect EDL properties and whether interfacial electrochemistry can be explained by a modified GCS model or an alternative model will have to be used.
- *In situ* spectroscopies will undoubtedly play an important role in answering some of the above questions. However, we have shown here that surface electrochemistry at this interface is highly sensitive to experimental conditions such as water content and whether or not the experiment is performed in a glovebox. For *in situ* spectroscopies to be meaningful they should ideally be performed in a highly controlled environment inside the glovebox. When this is not possible, detailed studies of the effect of the less controlled conditions (e.g., exposure of the electrolyte to the atmosphere for shorter or longer periods) on the surface electrochemistry will be needed.
- The generality of the formation of adsorbed layers for other nonaqueous interfaces likely depends on the chemical interactions between each metal/solvent pair. While the interphase layer observed here may be similar to the well-known SEIs in battery type systems, it forms at significantly less extreme potentials. Similar studies at various model interfaces will be needed to gain a more detailed understanding of the electrochemistry and formation mechanism of the interphase layer. Such studies will also help to assess the extent of similarity between the adsorbed layer detected here and battery-type SEI layers.

## Supplementary Material



## Notes

Detailed experimental procedures, further details relating to cleanliness and exclusion of water, additional aspects of CV analysis and additional information about XPS measurements (PDF)
