# Supplementary material for: Surface Electrochemistry of Au(111) in Acetonitrile Based Electrolytes: Formation of a Solvent Related Adsorbed Layer
Source: J Phys Chem Lett. 2026 Jul 8;17(29):8403–10. doi: 10.1021/acs.jpclett.6c01308 (PMC13403299; doi:10.1021/acs.jpclett.6c01308)
Supplement: Supplementary file 1 [file jz6c01308_si_001.pdf]

Supporting Information to

Surface Electrochemistry of Au(111) in Acetonitrile Based Electrolytes: Formation of a Solvent Related Adsorbed Layer

Greta P. Grossman[1], Milena Martins[2], Alenka Krizan[2], Andrijana Marojevic[2,3], Jan Bitenc[2], Dusan Strmcnik[2], Marc T. M. Koper[1]

[1] Leiden Institute of Chemistry, Leiden University, Einsteinweg 55, 2333 CC Leiden, the Netherlands, m.koper@chem.leidenuniv.nl
[2] National Institute of Chemistry, Hajdrihova 19, 1000 Ljubljana, Slovenia, *dusan.strmcnik@ki.si*
[3] Faculty of Chemistry and Chemical Technology, University of Ljubljana, Večna pot 113, 1000 Ljubljana, Slovenia

Supplementary Note 1: Experimental Methods

Acetonitrile (Sigma Aldrich, 99.999%, trace metal basis, electronic grade) was dried with 3 Å molecular sieves for 5 days and purified by fractional distillation inside an Ar filled glovebox. The amount of water was determined by Karl Fischer titration (Mettler Toledo, C10S Coulometric KF Titrator) to be < 1 ppm. Purity of the solvent was verified by gas chromatography mass spectrometry (GC-MS) (Figure S1). All salts were used without additional purification but were dried before use in a mini vacuum oven (Buchi Glass Oven G-300) inside the glovebox: $TBAClO_4$ (Sigma Aldrich, > 99%) at 80 °C for 5 days, $LiClO_4$ (Sigma Aldrich, 99.99%) at 150 °C for 5 days, $TBAPF_6$ (Sigma Aldrich, >99%) at 90 °C for 5 days. A Au(111) single crystalline disk (99.999%, MaTeck, 0.126 cm^2 geometric surface area) working electrode and Au wire (99.99%, MaTeck) counter electrodes were used for all electrochemical measurements. The Au single crystal and wires were flame annealed with a butane torch until a faint orange glow was observed and this glow was maintained for ~ 2 min by passing the Au crystal or wires in and out of the flame to avoid heating above the melting point. After this the electrodes were left to cool in air for a few seconds before transferring them to Ar atmosphere of the glovebox. The Au single crystal working electrode was transferred without placing it under vacuum. The annealing procedure for the Au(111) single crystal was tested by recording CVs in aqueous $HClO_4$ and H_2SO_4 solutions, which were found to correspond well to literature,[1] demonstrating that the Au(111) surface was well ordered with no significant contributions from defect sites. The reference electrode was a Ag wire (99.9%, Basi) used as a pseudo reference electrode. The Ag wire was polished with sandpaper and then rinsed with isopropanol followed by ultrapure water (Milli-Q, 18.2 MΩ cm) before each experiment and placed directly inside the Luggin capillary.

All glassware was cleaned prior to experiments by placing it overnight in a solution of 1 g/L $KMnO_4$ with 0.5 M H_2SO_4, followed by placing it for 30 minutes into a dilute piranha solution containing approximately 30 mM H_2SO_4 and 60 mM H_2O_2. This was followed by rinsing several times and boiling in ultrapure water (Milli-Q, 18.2 MΩ cm) at least 5 times. All glassware was then dried overnight in a vacuum oven at 150 °C and 100 mbar and then transferred to an Ar (Linde 5.0, ≥99.999%) filled glovebox with 0.1 < ppm H_2O and O_2 equipped with a solvent trap. All experiments were performed inside the glovebox, unless mentioned otherwise. It is important to note that great care was taken to ensure that a high standard of cleanliness was continuously maintained in the

glovebox and the vacuum oven used. Only pre-cleaned equipment was placed into each of these and chemical containers that were opened for any duration of time were limited to the ones used in these experiments. Before starting these experiments the glovebox atmosphere was renewed by three cycles or Ar purging (for 10 minutes with at least 30 minutes of Ar circulation through the solvent trap in between) to ensure no solvent residues remained in the atmosphere from previous experiments.

For experiments done outside of the glovebox all electrodes were prepared in the same way. Glassware was cleaned in the same way without the drying step. $TBAClO_4$ was used as received without additional drying. To ensure oxygen free conditions the cell was purged with Ar flow in the headspace and through the solution for 30 minutes. Ar flow in the headspace was maintained during the experiments. For the aqueous experiment in Figure 3 of the main text a reversible hydrogen reference electrode was used (Hydroflex, Gaskatel).

A three electrode cell was used with a shunt capacitor connecting the reference electrode to an additional gold wire electrode. The working electrode was connected in a hanging meniscus configuration. The reference electrode was placed inside a Luggin capillary to ensure that the point where the reference electrode senses the solution potential is close to the working electrode surface, reducing the Ohmic drop. All electrochemical measurements were performed using a PalmSens4 potentiostat. The starting potential of the CV was applied to the working electrode before immersion in the electrolyte so that it was immersed under potential control. The time between immersion and the start of the CV potential scan was kept at 30 seconds unless specified otherwise. The starting potential for ACN based CVs was 0.2 V vs Ag/Ag^+, with the exception of the CVs in Fig 1a), S2, S5, S10b) and S10 d) which were started at -0.4 V vs Ag/Ag^+ and Fig 1b), S6, S7 and S9a) which were started at 0.4 V vs Ag/Ag^+. See Figure S10 and accompanying discussion on the effect of starting potentials on the CV. The solution resistance was determined by electrochemical impedance spectroscopy (EIS) and 85% was compensated during the measurement within the potentiostat software. EIS was measured at the starting potential of each CV between 1 Hz and 10 000 kHz with an amplitude of 5 mV. The measured value of solution resistance was approximately 10 000 Ω for all of our measurements. Considering the high value of the solution resistance, the 85% compensation during the measurement was essential to ensure a linear effective scan rate. Without this compensation, the peaks appear significantly less sharp. We also note that the EIS measurements were performed after the CV to ensure that electrochemical conditioning before the start of the CV was limited to the application of the starting potential for a controlled duration of time, as mentioned above. We found that the small variation in solution resistance between measurements did not have any significant effect on the results.

For experiments in Figure 2 of the main text, water and salt additives were introduced directly into the cell through the opening for the working electrode, while the working electrode was not present. The solution was then left with Ar bubbling through the solution for ~ 5 min, which was sufficient to achieve a uniform distribution of additives due to the small solution volume (9 mL). Repetitions of certain CVs with additives with a longer mixing period showed no noticeable change. After the solution was mixed, the working electrode was freshly annealed, added to the cell and the meniscus was newly formed before starting the measurement of each CV trace. To introduce water into the cell under glovebox conditions, ~ 3 mL of ultrapure water was purged with Ar bubbling for ~ 10 min in a small vial, and the vial was quickly sealed so that the headspace was filled with mainly Ar. This vial was introduced into the glovebox without placing it under vacuum and was opened for the minimal required time to take small amounts of water using a micropipette

which were then added to the ACN cell. No change was seen in the O_2 and H_2O levels in the glovebox atmosphere during this procedure.

XPS measurements were carried out using a PHI 5000 VersaProbe III equipped with a monochromatic Al-Kα X-ray source. Single crystal Au samples were mounted on the sample holder using double-sided conducting copper tape. The samples were transferred from the glovebox to the XPS instrument in a dedicated vessel that maintained an inert atmosphere. All spectra were collected using an X-ray beam with a diameter of 200 μm and with the charge neutralizer turned off. High-resolution spectra were recorded with pass energy of 55 eV and a step size of 0.1 eV. Survey spectra were collected with pass energy of 224 eV and a step size of 1.0 eV.

Supplementary Note 2: Details relating to Cleanliness of Experiments and Exclusion of Water

Supplementary Note 2.1 Purification of ACN

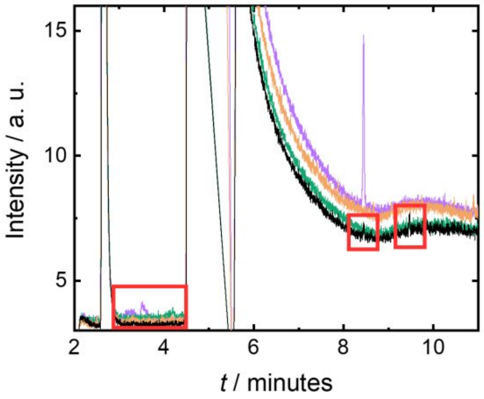

Figure S1 Gas chromatogram of the solvent as received (purple), after drying for 5 days with molecular sieves (black), after distillation of the dried solvent (green) and after electrochemistry (orange), impurity related peaks are shown by red frames, the chromatogram is cut off between ~4.5 – 5.5 min due to the ACN peak

The purity of ACN was characterised by GC-MS at different stages of the purification process, as shown in Figure S1. The as received solvent shows a large impurity peak around 8.5 min, which can tentatively be assigned to m-xylene based on mass spectrometry fragmentation. The smaller peaks between 3 – 3.5 min were identified as water and methanol, and were fully removed after treatment with molecular sieves. The larger impurity remains present after treatment with molecular sieves, but is removed with distillation. An additional small impurity (around 9.5 min) is introduced by drying with molecular sieves but is also removed with distillation. The orange trace shows the characterisation of the ACN electrolyte after electrochemical experiments. No additional impurities can be detected, indicating that no significant impurities or solvent decomposition products were introduced during experiments.

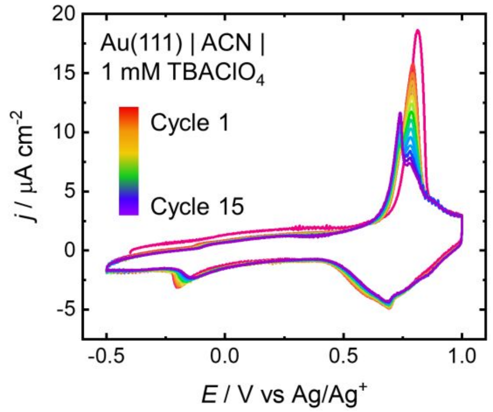

Figure S2 First 15 cycles of the CV of Au(111) in ACN with 1 mM $TBAClO_4$ at 50 mV s^{-1} with the same experimental parameters as the CV in Figure 1a) of the main text, but with a different batch of the distilled solvent

Although GC-MS spectra show that distillation was fully effective in removing impurities from the solvent, a small difference in electrochemical behaviour is noticeable between different batches of distilled ACN, as shown in Figure S2. Presumably this difference is due to differing impurity content, however, these impurities are below the detection limit of the GC-MS analysis. This finding suggests that electrochemical experiments are more sensitive to impurities than GC-MS analysis. In order to fully understand the effect of these small impurities on electrochemistry, alternative analysis methods are needed as well as advanced purification processes. However, these are outside the scope of our study, and considering that the differences between Figure S2 and Figure 1a) of the main text are extremely small, we can assume that electrochemical features discussed here do not originate from impurities.

Supplementary Note 2.2 CVs of Au(111) in ACN with 1 mM $TBAClO_4$ with Different Reference Electrodes

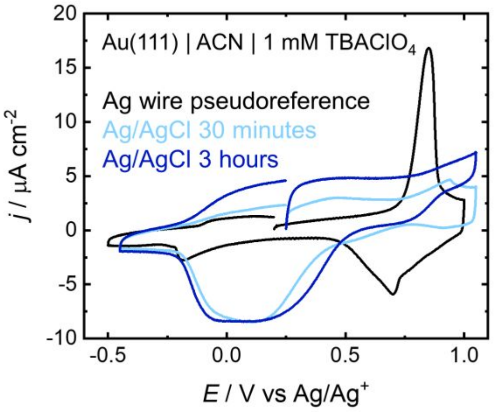

Figure S3 First cycles of the CV of Au(111) in ACN with 1 mM $TBAClO_4$ at 50 mVs^{-1} with an Ag wire reference electrode (black) and, in the same cell, with a leakless Ag/AgCl reference electrode 30 minutes (light blue) and 3 hours (dark blue) after immersion of the Ag/AgCl reference electrode in the solution

Figure S3 shows the changes in the CV when using a leakless Ag/AgCl electrode (eDAQ, ET069-1 leakless Ag/AgCl reference electrode) compared to a Ag wire pseudo reference electrode. The main feature visible in the CVs with the Ag/AgCl reference electrodes is a large reduction peak

that we tentatively assign to chloride desorption. The CV region negative of this peak, below ~ -0.2 V vs Ag/Ag$^+$ appears similar with the two reference electrodes, while the potential regions positive of this peak are significantly distorted with the Ag/AgCl reference electrode. We can tentatively interpret this behaviour by considering that positive of -0.2 V vs Ag/Ag$^+$ the electrode surface is covered with adsorbed chloride, which blocks other electrode processes such as the ACN related peak at 0.87 V vs Ag/Ag$^+$. We have found similar distortions in the CV when using leakless Ag/AgCl reference electrodes regardless of storage solution or the amount of time the particular reference electrode had been in use. We note however, that the amount of leakage likely depends on the solubility of the KCl filling solution in the solvent being used, therefore it is expected to differ between different solvents.

Supplementary Note 2.3 Impact of Cleanliness and Experimental Conditions on the CV of Au(111) in ACN with 1 mM TBAClO$_4$

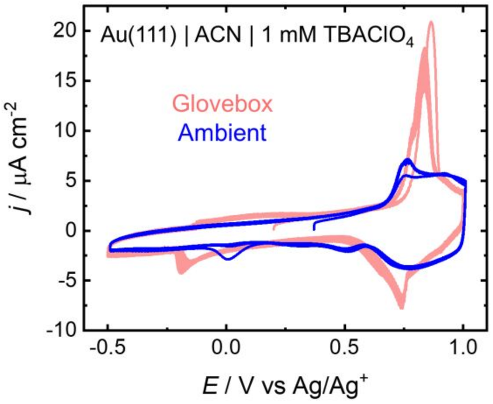

Figure S4 First 15 cycles of the CV of Au(111) in ACN with 1 mM TBAClO$_4$ at 50 mV s^{-1} inside the glovebox with a pre-cleaned solvent (pale red) and outside of the glovebox with the same pre-cleaned solvent (blue)

Figure S4 shows a comparison between the CVs obtained when performing the experiment in the glovebox with 1000 ppm added water (see Figure 2c of the main text) and on the benchtop in ambient conditions where we measured 750 ppm water after the experiment. All parameters were kept to be the same as far as was possible; the same chemicals and electrodes were used for both experiments and experimental equipment and electrodes were cleaned in the same way. The same distilled ACN solvent was used in both cases, but for the ambient experiment the solvent bottle had to be opened to air, which lead to elevated water content and possibly further contamination from the atmosphere. Water content in the purified ACN was measured as < 1 ppm under glovebox conditions. After the first time the bottle was opened to the atmosphere water content was measured as 150 ppm and it increased steadily each time the solvent was used in ambient conditions. Since the experiment shown in Figure S4 was performed after the ACN had already been stored outside of the glovebox for several weeks, the water content was likely already significantly elevated before the start of the experiment. We also expect that some additional water accumulated in the ACN due to exposure to the atmosphere while the experiment was set up and performed. To ensure an Ar atmosphere for the ambient experiment the electrochemical cell was purged with Ar in the headspace and through the solution before starting the experiment and Ar purging in the headspace was maintained throughout the experiment.

Between the CV done in the glovebox and in ambient conditions the same features can be observed: a sharp oxidation peak with a corresponding wider reduction peak around 0.8 V vs Ag/Ag^+, a smaller reduction peak around – 0.25 V vs Ag/Ag^+ and an otherwise featureless flat region below 0.5 V vs Ag/Ag^+. There are however significant differences in the shapes and relative positions of the peaks. The main adsorption peak in the glovebox starts out sharp and then progressively decays while in ambient conditions this peak starts out smaller and only decays very slightly. The adsorption process we have identified occurs in both conditions as evidenced by the presence of the characteristic peaks, however the exact nature of the adsorption process is very sensitive to the conditions. The differences observed here cannot be explained by the differing water content alone, as evidenced by the CVs in Figures 2c) and 2d) of the main text, which show that CVs performed in the glovebox with lower and higher water contents still differ from the ambient CVs. There is no obvious difference in the chemical composition of these interfaces other than the slightly different water content, making the origin of the observed differences unclear.

We can speculate on a possible explanation based on the equilibration time that may be needed to reach a stable configuration of water molecules in the ACN solution. As stated in the main text (in the discussion of Figures 2c) and d)), ACN/water mixtures are often not homogeneous and a specific distribution of the molecules is thermodynamically more favourable, depending on the exact composition. It seems possible that with low water contents some equilibration time would be needed to reach a thermodynamically stable state due to the low density of water molecules. In our case, ACN stored outside the glovebox already had a relatively high water content before starting the experiment, so would have had time to equilibrate. On the other hand, in our water addition experiments performed in the glovebox, ACN solutions had water content < 1 ppm at the start of the experiment and water was added gradually, with CVs measured directly after each water addition. Thus, if equilibration is indeed slow, it may be that there wasn’t sufficient time for this in our glovebox measurements. Additional experimental studies will be needed to fully understand the origins of this difference. For example, a measurement where the cell is fully set up and sealed inside the glovebox and then only opened for a minimal period of time to attach external Ar lines for purging may be interesting. Such an experiment would help in detecting (the lack of) water and contaminants introduced when the electrolyte is exposed to the atmosphere for very short periods of time. Such studies will be valuable in connecting the idealised conditions in this study with more practically relevant ambient conditions.

Supplementary Note 3: Further Aspects of CV Analysis

Supplementary Note 3.1 Charge Analysis of the CV of Au(111) in ACN with 1 mM $TBAClO_4$

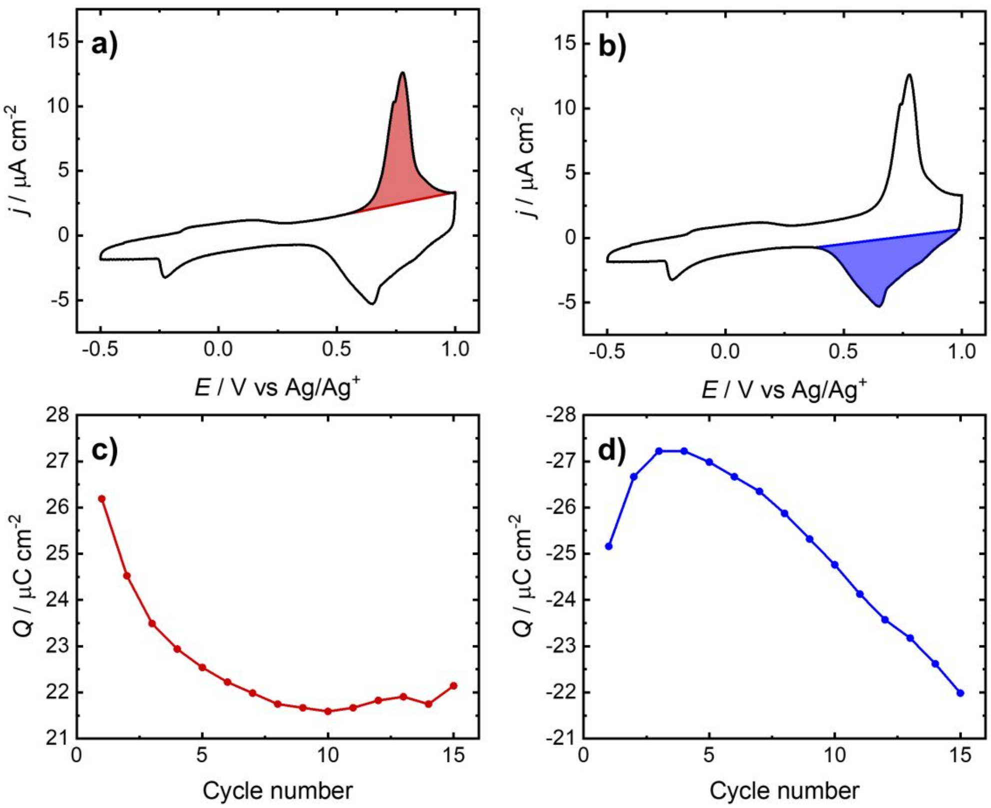

Figure S5 Charge density analysis of the CV in Figure 1a) of the main text, where a) and b) show baselines and areas used for the integration of the oxidative and reductive charges respectively, for cycle 3 as an example and c) and d) show the charge density in the oxidative and reductive scans respectively, as a function of cycle number

Figures S5a) and b) show a schematic representation of how the integration was performed to obtain the charge under the main oxidation and reduction peaks. Figure S5c) and d) show the charge under the oxidative and reductive current as a function of cycle number for the CV in Figure 1a) of the main text. It is difficult to choose a rational baseline for integrating the peaks, since the CV shows significant tilt and the peaks are not simple ideal processes. The tilt in the CV can likely be attributed to minor wetting of the edges of the electrode, which can manifest as an additional parallel resistive component. However, we also cannot fully exclude the presence of an impurity related faradaic process. Considering these issues, the baseline cannot be precisely chosen and the charge under the peaks can only be determined approximately. To be able to determine an approximate charge, a simple tilted straight-line background was used to account for the tilt. To ensure that comparison between subsequent CV cycles is still valid, the same straight baseline was used for each CV cycle, with the exception of the first oxidative peak, which has a significantly different background current (see Figure 1a in the main text). This procedure will include non-trivial errors in the charge determination, however, obtained charge values can be used in an approximate way. By assuming that the errors in each CV cycle are similar, trends over cycling can be safely interpreted. However, absolute charge values can only be interpreted in an

approximate sense. Oxidative and reductive charges cannot be directly compared, since the different baselines chosen for these features presumably have different errors.

There are some deviations from the overall decreasing trend in the charge density mentioned in the main text, which we can explain as follows. The decreasing trend is not followed for the first two reductive cycles, which have charges less negative than expected, indicating that the extent of the desorption might be lower for these initial cycles, when the total amount of adsorbed species is assumed to be smaller. The last five oxidative cycles also show slightly more positive than expected charges which do not align with the trend. This increasing region corresponds to the transition of the large oxidative peak at 0.87 V vs Ag/Ag^+ to an overlapping sharp peak around 0.74 V vs Ag/Ag^+ and at the same time the emergence of a small flat oxidative feature between [0.80; 0.95] V vs Ag/Ag^+.

Supplementary Note 3.2 Scan Rate Dependent Parameters of the CV of Au(111) in ACN with 1 mM $TBAClO_4$

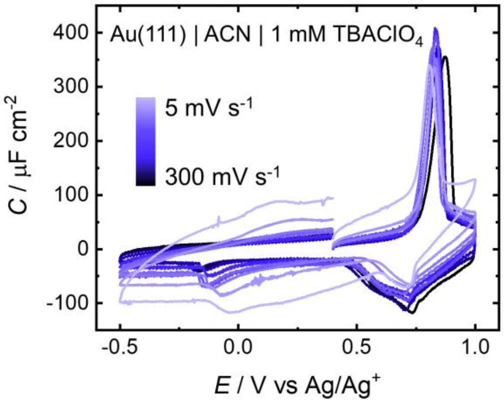

Figure S6 Capacitance calculated from the first CV cycle at various scan rates for Au(111) in ACN with 1 mM $TBAClO_4$

Figure S6 shows CV curves measured at different scan rates, normalised by the scan rate in each case. The curves were measured in a random order to exclude convolution from time effects. When CV curves were re-measured in a different order there was no change in their appearance.

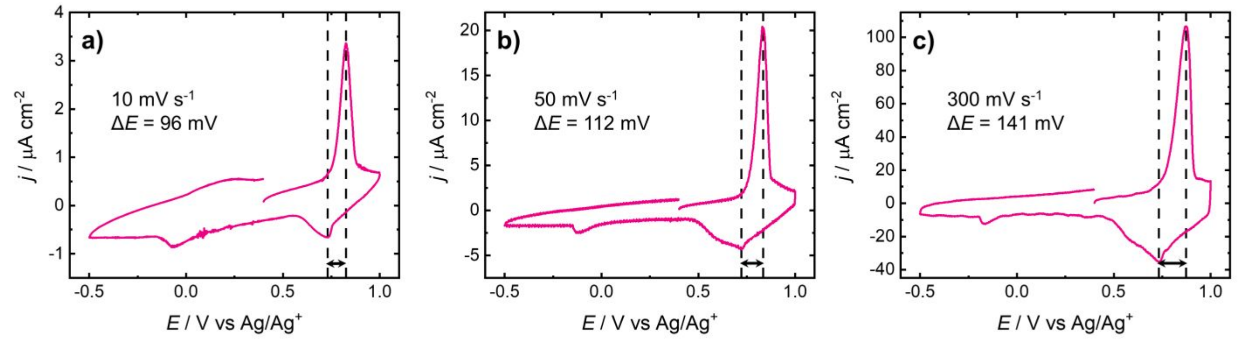

Figure S7 Example of peak to peak separation values for the main adsorption peak in the CV of Au(111) in ACN with 1 mM $TBAClO_4$ at three different scan rates, the measured peak positions are indicated by dashed lines

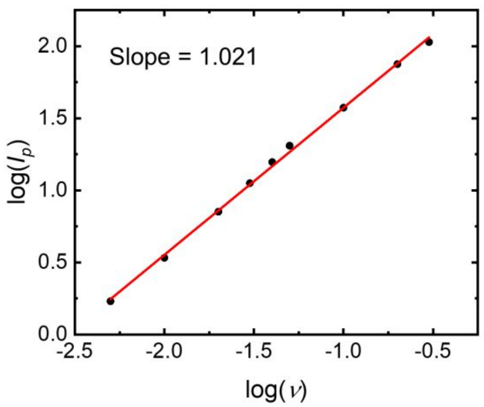

Figure S8 Peak current for the large oxidation peak at 0.87 V vs Ag/Ag⁺ versus the scan rate plotted on a log-log scale (black dots) and linear fit (red line), with all values measured for the first cycle of the CV of Au(111) in ACN with 1 mM $TBAClO_4$ with different scan rates (Figure S6), the R^2 value was calculated as 0.99813 and the slope as 1.021

Supplementary Note 3.3 Comparison Between the Butterfly Peaks of Au(111) in H_2SO_4 and the Sharp Peaks Observed in the High Potential Region of the CV of Au(111) in ACN with 1 mM $TBAClO_4$

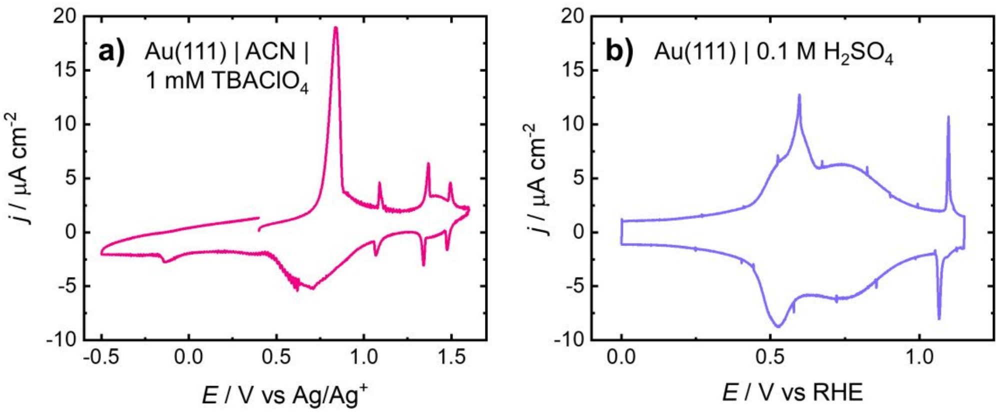

Figure S9 First cycle of the CV of Au(111) at 50 mV s^{-1} a) in ACN with 1 mM $TBAClO_4$ between [-0.5;1.5] V vs Ag/Ag^+ and b) in 0.1 M H_2SO_4

Table S1 Comparison of the peak shapes, positions and reversibility between the butterfly peaks of Au(111) in H_2SO_4 and the sharp peaks observed in the high potential region of Au(111) in ACN with 1 mM $TBAClO_4$ with the relative position of the peaks measured versus the main adsorption peak in each case (0.87 V vs Ag/Ag^+ for ACN and 0.6 V vs RHE for SO_4^{2-})

	SO_4^{2-}	ACN 1	ACN 2	ACN 3
Peak to peak separation / mV	31	21	28	18
FWHM / mV	6	12	15	15
Relative position /mV	502	250	530	660

Supplementary Note 3.4 Effect of the Starting Potential on the CV of Au(111) in ACN with 1 mM $TBAClO_4$

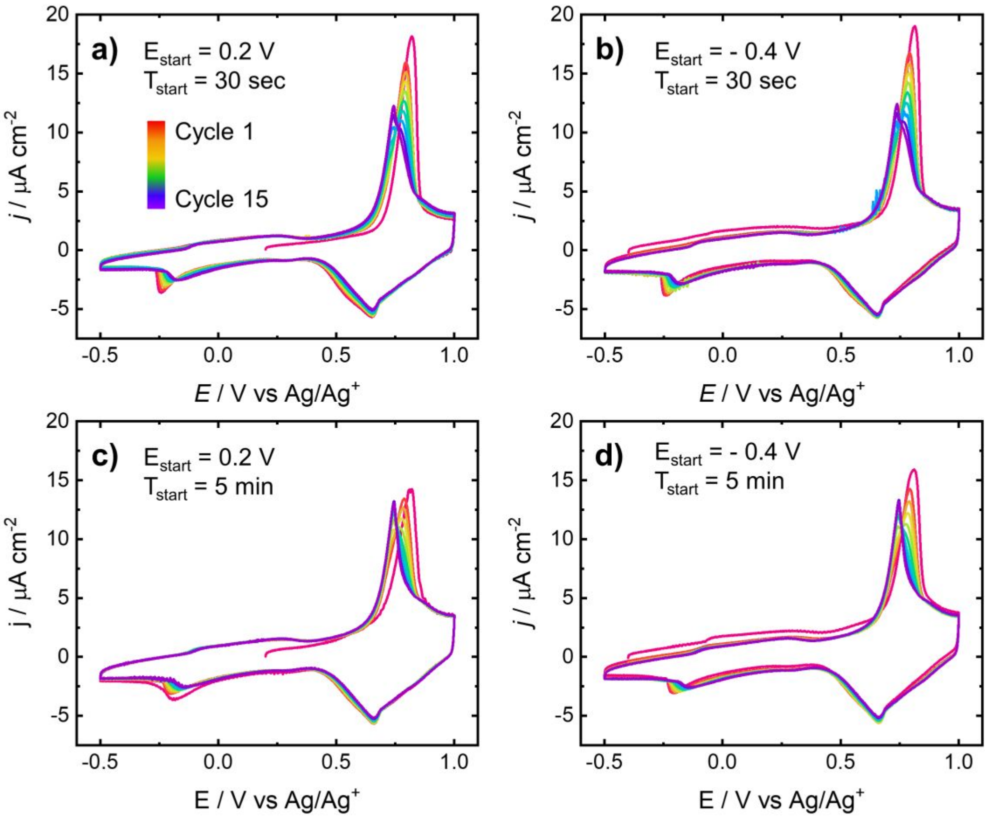

Figure S10 First 15 cycles CV of Au(111) in ACN with 1 mM $TBAClO_4$ at 50 mV s^{-1} with a starting potential of 0.2 V vs Ag/Ag^+ (a) and c)) or -0.4 V vs Ag/Ag^+ (b) and d)) held for 30 seconds (a) and b)) or 5 minutes (b) and d))

Figure S10 shows the effect of two different starting potentials held for different amounts of time on the CV features. When the starting potential is held for 30 seconds, CVs with different starting potentials appear similar, which could lead to the conclusion that the starting potential has no effect on the CV features. However, when the starting potentials are held for 5 minutes, slight differences are detectable in the CVs, where in the first cycle of the CV with starting potential of 0.2 V vs Ag/Ag^+ the main oxidation peak is slightly lower and the reduction peak at – 0.25 V vs Ag/Ag^+ is wider and larger. The difference between different starting potentials imply that in the 30 second holding time before the CV (Supplementary Note 1), electrochemical processes have already started happening, and the electrode surface is beginning to be modified. This initial modification proceeds slightly differently at different potentials, resulting in a slightly different surface and giving rise to slightly different electrochemical features. These differences are present already when the starting potentials are held for 30 seconds, but they become more obvious when the starting potentials are held for 5 minutes.

Supplementary Note 4: Full CVs with Various Electrolytes

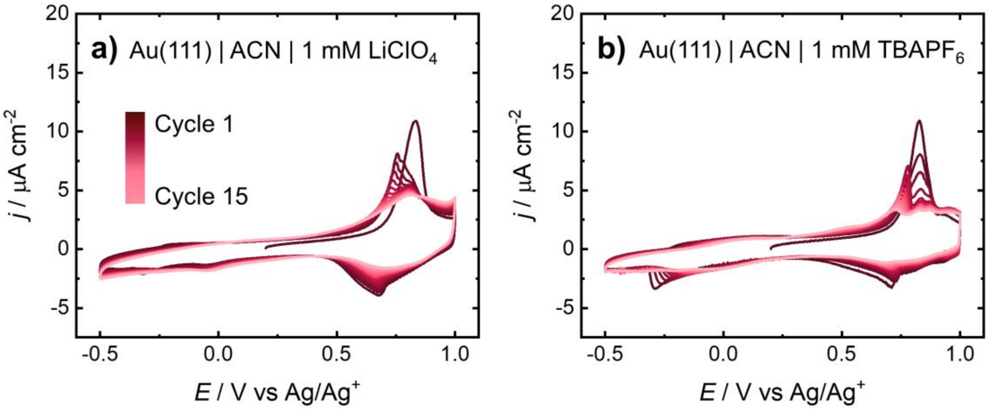

Figure S11 First 15 cycles of the CV of Au(111) in ACN with a) 1 mM $LiClO_4$ and b) 1 mM $TBAPF_6$ at 50 mV s^{-1}

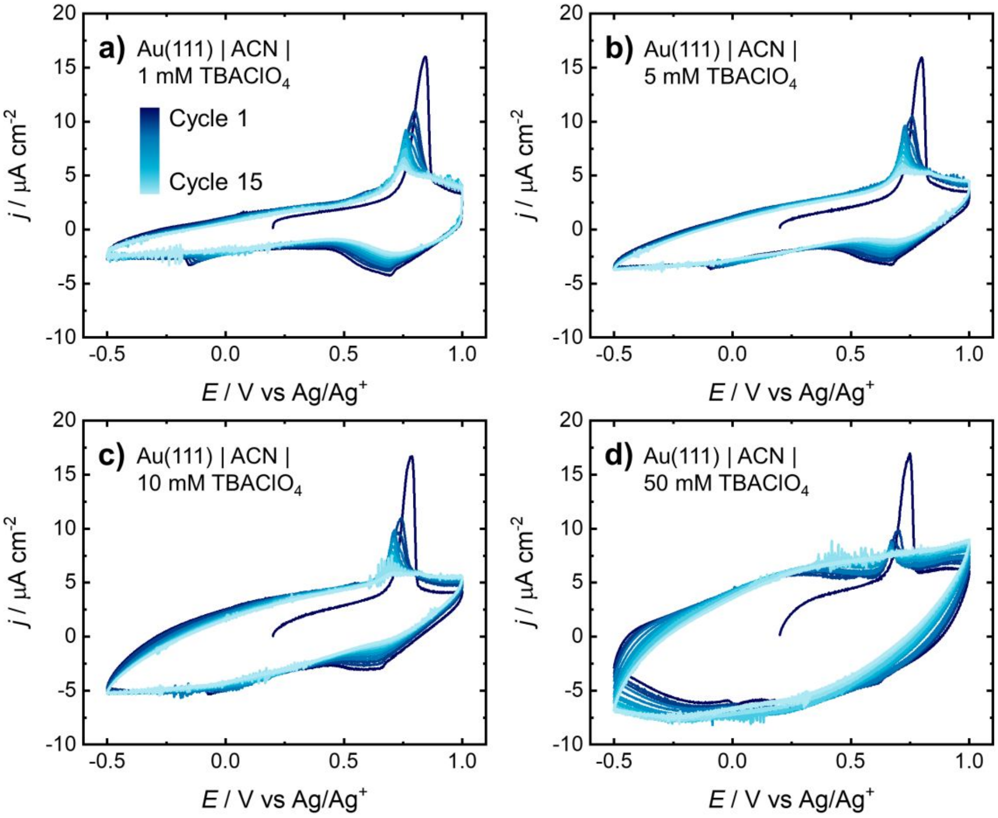

Figure S12 First 15 cycles of the CV of Au (111) in ACN with a) 1, b) 5, c) 10 and d) 50 mM $TBAClO_4$ at 50 mV s^{-1}

Supplementary Note 5: Additional Information about XPS Measurements

Figure S13 below shows the CV of Au(111) in ACN with 1 mM $LiClO_4$ taken right before placing the sample in the XPS chamber. After electrochemical treatment and washing in the glovebox, the Au(111) sample was transferred to the XPS chamber under a continuously maintained Ar atmosphere.

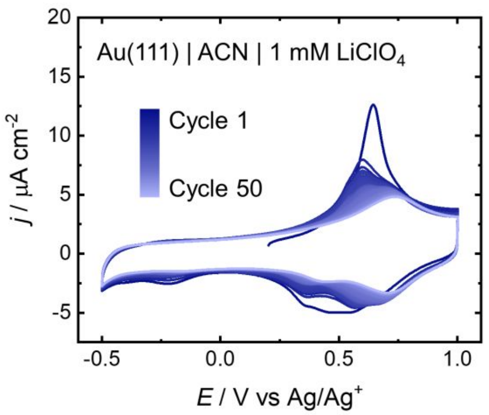

Figure S13 First 50 cycles of the CV of Au(111) in ACN with 1 mM $LiClO_4$, 50 mV s^{-1}, taken directly before the XPS measurement

In Figure S14 below survey spectra taken at three or four different spots on all four measured samples are shown. Region scans were taken and analysed of the C 1s, O 1s and Au 4f core levels, however these signals are strongly affected by adventitious carbon, which is a ubiquitous contamination in XPS when not done fully under ultra-high vacuum conditions.[2–4] The shape of the C 1s and O 1s signals agreed well with expectations based on the species commonly found in adventitious carbon and the ratios between the C 1s and O 1s species also approximately aligned with expectations based on adventitious carbon species.[2–4] Based on the normalised areas of the XPS peaks the amount of adventitious carbon present in our sample is higher than the amount of the solvent related adsorbed layer. Due to this reason and since we are unsure of the exact binding energies expected both for the adsorbed layer and for the adventitious carbon species, we cannot deconvolute signals from adventitious carbon and from the solvent related adsorbed layer in the case of the C 1s and O 1s spectra. Additionally, since the amount of adventitious carbon is so high in comparison to the adsorbed layer, it is also mainly the amount of adventitious carbon on each sample that influences the areas of the Au 4f peaks (which are expected to decrease if there are more chemical species on top of the Au sample). Thus, we also cannot use the Au 4f signals in our analysis. Li 1s signals also cannot be used due to overlap with the Au 5p signal, leaving the N 1s and Cl 2p core levels as ones that can provide meaningful data for our analysis. These signals are not affected by adventitious carbon or other common contaminants and provide insight into the presence of both ACN and $LiClO_4$.

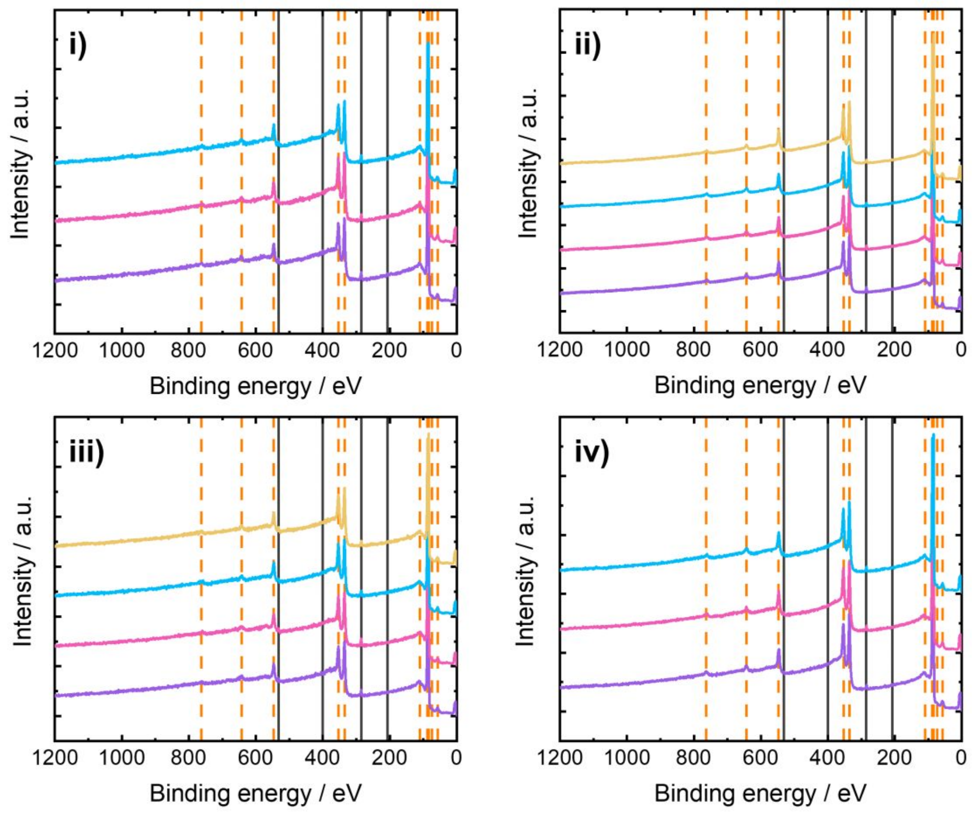

Figure S14 XPS survey spectra of three or four spots on samples i) -iv), the expected positions for the Au core levels are labelled with orange dashed lines and expected positions for the core levels of other expected elements are labelled in grey

All analysis of the XPS spectra was performed using the CasaXPS software.[5] The following procedure was followed for analysis:

1. Energy calibration was performed by specifying the location of the Au 4f peak at 84 eV.[6]
2. Background subtraction was performed for all region scans using a linear background, which was justified since there was no visible energy step in the background signal.[7]
3. Peak fitting was performed using the GL(30) lineshape in CasaXPS. For the N 1s core level two component peaks were added and the full width half maximum (FWHM) values were constrained to be equal for the two components. No further constraints were applied. For the Cl 2p core level two component peaks were added for each of the two observed chemical states to account for the spin-orbit splitting expected for this core level. FWHM values were constrained to be the same, area ratios were constrained as 1:2 and peak to peak distance was constrained to be 1.6 eV[6] for the two component peaks of each of the doublets.
4. Peak position and FWHM values were measured for the Gaussian-Lorentzian curves obtained from the fitted peak models.
5. The raw areas were taken as the area between the synthetic peak model and the fitted background. To obtain normalised values these areas were divided by the photoelectron

cross section, the attenuation length and the transmission function expected for each core level to account for the differing sensitivity of the different core levels.

Figures S15 – S18 below show the N 1s and Cl 2p region scans at three or four different spots on samples i) – iv). We note that spectra for the freshly annealed sample iv) (Figure S18) have higher signal-to-noise levels due to the shorter time spent collecting photoelectrons at a single data point. Specifically, the time per data point of N 1s and Cl 2p spectra collected for samples i) – iii) was 16 s/data point while for sample iv) treatment this was only 1.6 s/ data point. Despite the higher signal-to-noise ratio for sample iv) it can clearly be seen that there are no peaks in the two spectral regions shown. Longer collection times would decrease the signal-to-noise levels for this sample. However, since we do not expect sample iv) to contain any N or Cl containing species, and considering practical limitations on measurement time, the shorter data collection times were judged to be sufficient to confirm the absence of these species.

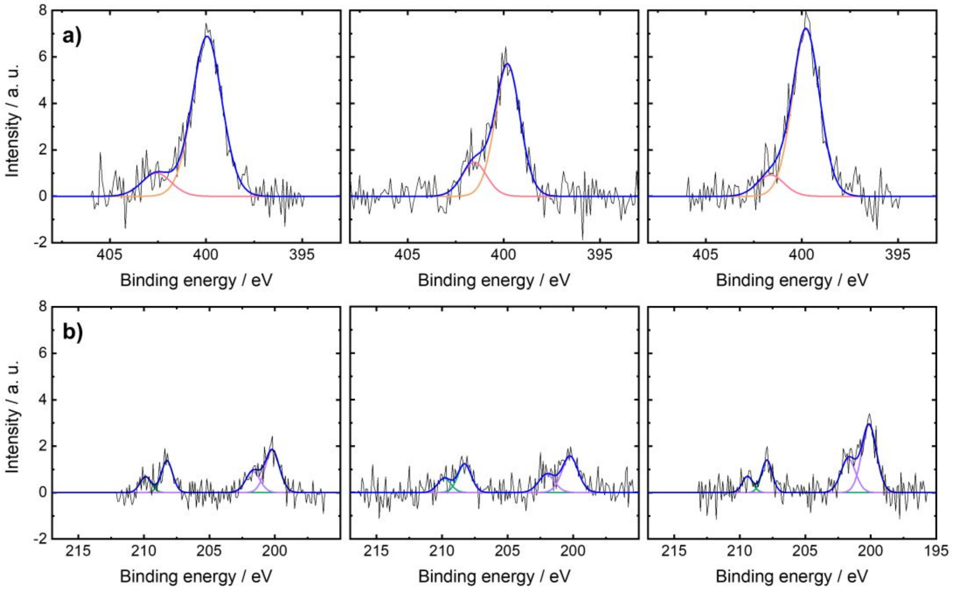

Figure S15 Region scans of Au(111) cycled electrochemically (sample i)) at three different spots a) in the N 1 s region, with the fitted data envelope shown in blue and the component peaks in pink and orange and b) in the Cl 2 p region with the fitted data envelope shown in blue and component peaks in green and purple

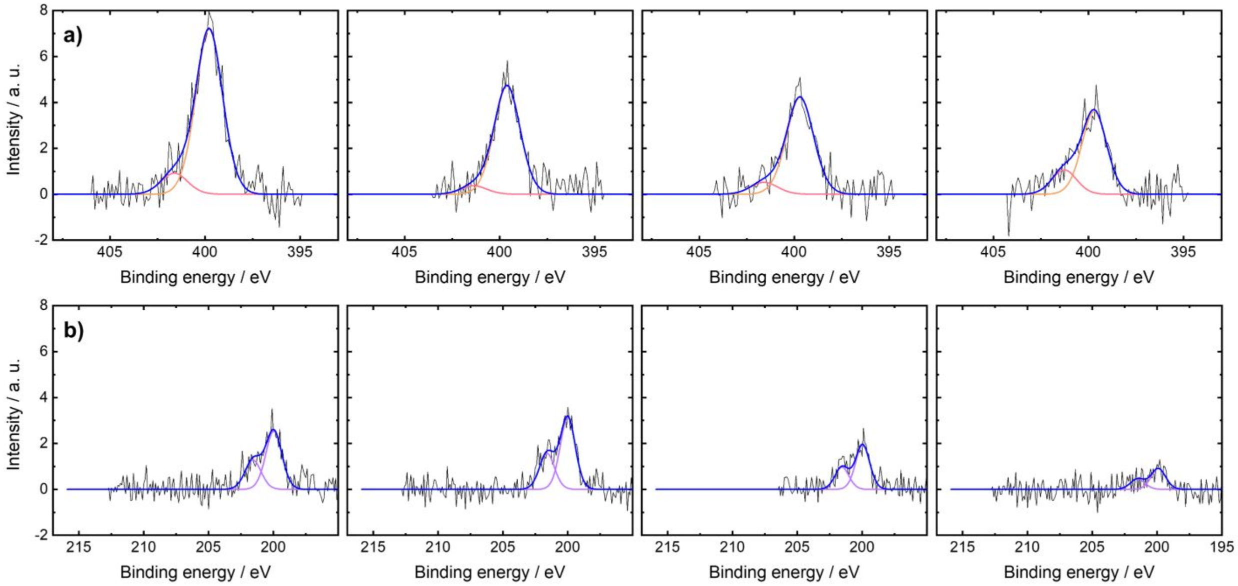

Figure S16 Region scans of Au(111) held at 0.2 V (sample ii)) at four different spots a) in the N 1 s region, with the fitted data envelope shown in blue and the component peaks in pink and orange and b) in the Cl 2 p region with the fitted data envelope shown in blue and component peaks in purple

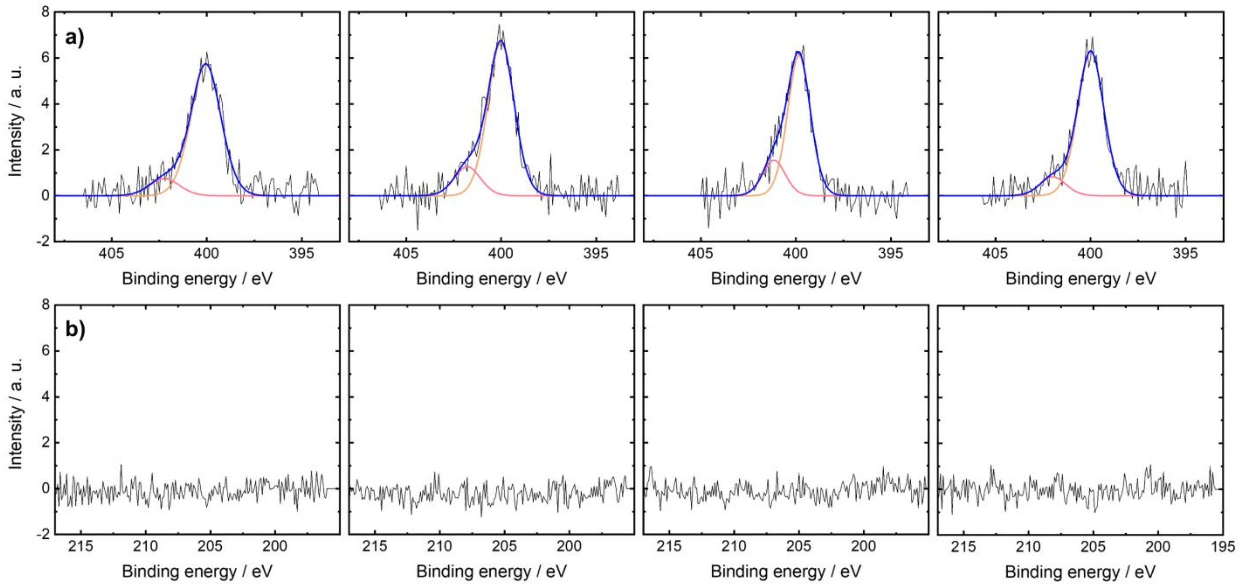

Figure S17 Region scans of Au(111) immersed in the electrolyte at OCP (sample iii)) at four different spots a) in the N 1 s region, with the fitted data envelope shown in blue and the component peaks in pink and orange and b) in the Cl 2 p region

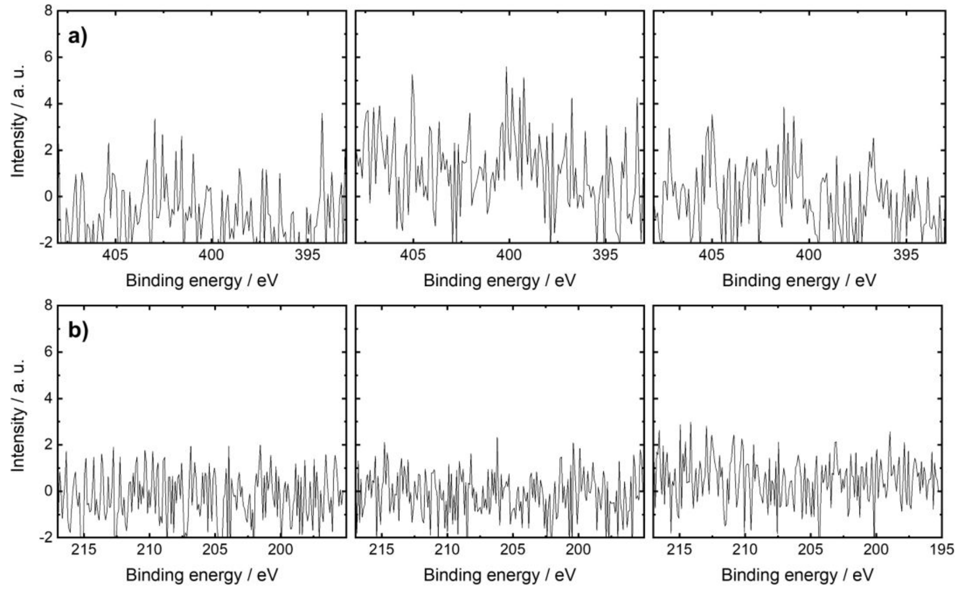

Figure S18 Region scans of the freshly annealed Au(111) (sample iv)) at three different spots a) in the N 1 s region and b) in the Cl 2 p region

Table S2 Characteristic values obtained for XPS spectra of samples i) – iii) taken as average values from all spots on each sample, the error is taken as the standard deviation

		Position/ eV		FWHM/ eV	Area/ a. u.
N 1s	i)	A	399.84 ± 0.09	1.7 ± 0.1	393 ± 45
		B	401.9 ± 0.6		
	ii)	A	399.66 ± 0.04	1.6 ± 0.1	257 ± 40
		B	401.3 ± 0.2		
	iii)	A	399.97 ± 0.09	1.6 ± 0.2	361 ± 29
		B	401.7 ± 0.9		
Cl 2 p comp. C	i)	208 ± 0.2		1.0 ± 0.1	44 ± 2
Cl 2 p comp. D	i)	200.19 ± 0.06		1.35 ± 0.08	89 ± 29
	ii)	199.96 ± 0.04		1.36 ± 0.08	93 ± 44

Supplementary References

1 H. Angerstein-Kozlowska, B. E. Conway, A. Hamelin and L. Stoicoviciu, Elementary steps of electrochemical oxidation of single-crystal planes of Au Part II. A chemical and structural basis of oxidation of the (111) plane, *Journal of Electroanalytical Chemistry and Interfacial Electrochemistry*, 1987, **228**, 429–453.

2 G. H. Major, N. Fairley, P. M. A. Sherwood, M. R. Linford, J. Terry, V. Fernandez and K. Artyushkova, Practical guide for curve fitting in x-ray photoelectron spectroscopy, *Journal of Vacuum Science & Technology A*, 2020, **38**, 061203.

3 T. R. Gengenbach, G. H. Major, M. R. Linford and C. D. Easton, Practical guides for x-ray photoelectron spectroscopy (XPS): Interpreting the carbon 1s spectrum, *Journal of Vacuum Science & Technology A*, 2021, **39**, 013204.

4 J. D. Henderson, B. P. Payne, N. S. McIntyre and M. C. Biesinger, Enhancing Oxygen Spectra Interpretation by Calculating Oxygen Linked to Adventitious Carbon, *Surface and Interface Analysis*, 2025, **57**, 214–220.
5 www.casaxps.com
6 J. F. Moulder, *Handbook of X-ray Photoelectron Spectroscopy: A Reference Book of Standard Spectra for Identification and Interpretation of XPS Data*, Physical Electronics Division, Perkin-Elmer Corporation, 1992.
7 M. H. Engelhard, D. R. Baer, A. Herrera-Gomez and P. M. A. Sherwood, Introductory guide to backgrounds in XPS spectra and their impact on determining peak intensities, *Journal of Vacuum Science & Technology A: Vacuum, Surfaces, and Films*, 2020, **38**, 063203.
